# Supplementary material for: Serum IgG titers against periodontal pathogens are associated with cerebral hemorrhage growth and 3-month outcome
Source: PLoS One. 2020 Oct 28;15(10):e0241205. doi: 10.1371/journal.pone.0241205 (PMC7592768; doi:10.1371/journal.pone.0241205)
Supplement: S1 File — (PDF) [file pone.0241205.s001.pdf]

Funding

This study was supported by research grants from Japan Society for the Promotion of Science KAKENHI (grant numbers 17K17350, 17K17907, and 18K10746).

Competing Interests

Hirofumi Maruyama reports research support from Eisai, Pfizer, Takeda Pharmaceutical, Otsuka Pharmaceutical, Nihon Pharmaceutical, Shionogi, Teijin Pharma, Fuji Film, Boehringer Ingelheim, Sumitomo Dainippon Pharma, Nihon Medi-Physics, Bayer, MSD, Daiichi Sankyo, Kyowa Hakko Kirin, Sanofi, Novartis, Kowa Pharmaceutical, Astellas Pharma, Tsumura, Japan Blood Products Organization, Mitsubishi Tanabe Pharma, and Mylan which are unrelated to the submitted work. This does not alter our adherence to *PLOS ONE* policies on sharing data and materials. All other authors declare that they have no conflicts of interest.
