# Supplementary material for: Serum IgG titers against periodontal pathogens are associated with cerebral hemorrhage growth and 3-month outcome
Source: PLoS One. 2020 Oct 28;15(10):e0241205. doi: 10.1371/journal.pone.0241205 (PMC7592768; doi:10.1371/journal.pone.0241205)
Supplement: Attachment — Submitted filename: Serum IgG periodontal pathogens and Cerebral Haemorrhage _ Reviewer .docx [file pone.0241205.s003.docx]

**Serum IgG titers of periodontal pathogens predict cerebral hemorrhage growth and 3-month outcome**

This article addresses the association between serum IgG titers of periodontal pathogens as predictors for cerebral hemorrhage growth and poor functional outcome at 3 months (i.e. modified Ranking Scale ≥3). The topic is interesting and it has not been explored before on a prospective cross-sectional study for cerebral hemorrhage. The abstract is appropriate for the content of the text. The authors mentioned relevant information regarding the ‘non-availability’ for reversal agents for oral anticoagulants by the time the study underwent, and their awareness for lack of periodontal disease clinical status from participants. Although the analysis included the most common vascular risk factors, there are some clinical characteristics that should have been considered to adjust during the statistical analysis, such as: Glasgow Coma Scale score, intraventricular hemorrhage extension, and early hematoma growth neuroimaging predictors (e.g. blend sign). Furthermore, since early blood pressure control in acute intracerebral hemorrhage is a key prognostic factor, it would have been useful to be mentioned if any of the participants required pharmacological blood pressure management during the first 24 hours, and consider to adjust for this on the statistical analysis. The conclusions that “elevated serum IgG titers of Aggregatibacter actinomycetemcomitans is associated to cerebral hemorrhage growth at 24 hours follow-up from admission and yet no effect on functional outcome at 3-months follow-up, and that elevated IgG titers of Fusobacterium nucleatum predicted a poor outcome”, are reasonable given the data reported in the article, but overreached the collected data.

Below there are more specific comments that should be clarified/addressed:

Major issues:

- P. 10: Please provide prevalence estimates regarding the IgG titers of periodontal pathogens in hemorrhagic stroke taken from the literature. More information needs to be added about the sample size calculation, statistical power, significance level and effect size. The sample size needs to be more extensively explained by the authors; this will allow to be precise about sizes effect (if any) in order to make solid conclusions and give opportunity to generate new hypothesis for future research.
- P. 12: Table 2. Select a more concise heading. It is not clear if the serum IgG titers makes reference to the ‘reference range’ from the five healthy volunteers or if these measurements are considered significant positive titers. It could be helpful to add a brief explanation on the ‘Results’ section.
- P. 14: Table 3. It would be more informative if you add to the ‘P values’ the odds ratio and confidence intervals for the univariate and multivariable analysis. Also, notice that data from the table considered four patients with history of atrial fibrillation and five patients taking anticoagulants on the positive hematoma growth group. On this same group, from 13 patients with positive hematoma growth, twelve had a hypertensive etiology, zero amyloid angiopathy and the remaining one participant is not mentioned on the table data. It is relevant that authors address this on the results section and verified data in this table.
- P. 17: Table 4. It would be more informative if you add to the ‘P values’ the odds ratio and confidence intervals for the univariate and multivariable analysis.

Minor issues:

1. Introduction:
- P. 5: Please add more background information about the potential mechanism proposed for the influence of periodontal disease in stroke since this will give more context to the reader. It is required more detail about the large cohort study that demonstrated the association between periodontal disease and the incidence of specific etiologies for stroke (i.e. include hazard ratio and confidence interval for these statements).
- P. 6: Regarding the aim of the study at the end of the introduction – ‘Therefore, to understand how’ – could be rephrase for – ‘to determine if’ – since the outcome is about prognosis and not pathophysiology.
  2. Materials and methods:
- P. 6: It is not clear all the inclusion and exclusion criteria for participants (e.g. >18 years-old, etiologies stated as ‘others’ cerebral hemorrhage causes).
- P. 7: Please confirm if participants underwent head computed tomography and/or magnetic resonance at admission, and please clarify who performed the NIHSS assessment.
- P. 7-8: It is worth to include the references for previously established definitions such as: ‘hypertension’ (e.g. American or European Hypertension Guidelines), ‘diabetes mellitus’, ‘dyslipidemia’, and ‘atrial fibrillation’; as there could be small differences on diagnostic criteria.
- P. 8: It is worth to explicitly state if the neuroimaging evaluation (i.e. hematoma volume measurements) remained blinded from the clinical assessment.
- P. 9: Regarding the determination of serum IgG titers, more detail about sampling and processing methods would be welcomed or they could be revisited as supplementary material.
  3. Discussion
- P. 16, line 3: It is recommended to unified the format ‘(listed in Tables 1 & 2, and cerebral hematoma growth)’ instead it could be referred as ‘(listed in Tables. 1, 2 and 3)’.
- P. 16: Limitations should be updated, including comments regarding sample size and others addressed at the beginning of this review.
  4. Title
- P. 1: Consider using a more accurate term such as ‘association’ or ‘relationship’ instead of ‘predict’. This would be more appropriate for the reader expectations.
